# Maternal Exposure to Low-Dose BDE-47 Induced Weight Gain and Impaired Insulin Sensitivity in the Offspring

**DOI:** 10.3390/ijms25168620

**Published:** 2024-08-07

**Authors:** Sandra Strunz, Rebecca Strachan, Mario Bauer, Ana C. Zenclussen, Beate Leppert, Kristin M. Junge, Tobias Polte

**Affiliations:** 1 Helmholtz Centre for Environmental Research—UFZ, Department of Environmental Immunology, 04318 Leipzig, Germanymario.bauer@ufz.de (M.B.); ana.zenclussen@ufz.de (A.C.Z.); kristin.junge@akad.de (K.M.J.); 2 Department of Dermatology, Venerology and Allergology, Leipzig University Medical Center, University of Leipzig, 04318 Leipzig, Germany; 3 School of Health and Social Sciences, AKAD University Stuttgart, 70191 Stuttgart, Germany

**Keywords:** BDE-47, overweight, metabolism, leptin, pregnancy, sex-specific effect, epigenetics

## Abstract

Polybrominated diphenyl ethers (PBDEs), commonly used as synthetic flame retardants, are present in a variety of consumer products, including electronics, polyurethane foams, textiles, and building materials. Initial evidence from epidemiological and experimental studies suggests that maternal PBDE exposure may be associated with a higher BMI in children, with disturbance of energy metabolism and an increased risk of Type 2 diabetes. However, the causality between early exposure to real-life PBDE concentrations and increased weight as well as mechanisms underlying impaired metabolic pathways in the offspring remain elusive. Here, using a mouse model we examined the effect of maternal exposure to 2,2′,4,4′-tetrabrominated diphenyl ether (BDE-47), the most abundant congener in human samples, on offspring weight gain and energy homeostasis using a mouse model. Maternal exposure to BDE-47 at low dose resulted in weight gain in female offspring together with an impaired glucose and insulin tolerance in both female and male mice. In vitro and in vivo data suggest increased adipogenesis induced by BDE-47, possibly mediated by DNA hypermethylation. Furthermore, mRNA data suggest that neuronal dysregulation of energy homeostasis, driven via a disturbed leptin signaling may contribute to the observed weight gain as well as impaired insulin and glucose tolerance.

## 1. Introduction

Childhood overweight and obesity has reached pandemic proportions in most developed countries and continues to increase worldwide. Recent reports suggest that overweight and obesity affect up to a third of the child population in Europe and North America [1,2]. In addition to genetic predisposition and high-caloric food intake, which is often associated with predominantly sedentary behavior and physical inactivity, environmental factors were found to contribute to and/or worsen the onset of overweight and obesity [3,4,5]. It has been shown that environmental influences like chemical exposures during critical time windows, such as fetal development, leads to disruptions in physiological, endocrine, and metabolic signaling, resulting in long-lasting health effects [6,7]. Epigenetic changes such as altered DNA methylation have been described to play a critical role as mediators between exposure and early developmental programming of disease [8,9]. Synthetic chemicals that disrupt the endocrine system are endocrine disrupting chemicals (EDCs), some of which, known as obesogens, have already been shown to contribute to developmental programming towards obesity following exposure in the perinatal period [9,10]. EDCs can be found in a variety of everyday products, to which they have been added, for example as plasticizers, preservatives, or stabilizers [11,12]. Exposure to EDCs is ubiquitous and inevitable. These chemicals can enter the body through food and water intake, skin absorption, or inhalation [4]. One group of chemicals with suspected obesogenic effects are polybrominated diphenyl ethers (PBDEs), which are widely used as synthetic flame retardants in consumer products, including electronics, polyurethane foams, textiles, and building materials [13], and are absorbed through, for example, contaminated food and drinking water [14].

There is initial evidence from epidemiological studies that maternal PBDE exposure may be associated with a higher BMI in children [15], with disturbance of energy metabolism and an increased risk of Type 2 diabetes [16]. Experimental studies in rat models have also shown that exposure to PBDEs may be associated with weight gain in offspring [17,18]. However, the mechanisms of early exposure to PBDEs leading to increased weight and impaired energy metabolism in offspring remain elusive.

In the present study, we examined the effects of maternal exposure to 2,2′,4,4′-tetrabromodiphenyl ether (BDE-47), the dominant congener in human samples, at a very low concentration relevant to daily life, on weight gain and metabolic parameters in the offspring. Using an in vivo mouse model, we hereby demonstrate that maternal exposure to BDE-47 resulted in weight gain in females and impaired glucose and insulin tolerance in both female and male offspring. The weight gain induced by BDE-47 was associated with increased adipogenesis mediated by DNA hypermethylation. Furthermore, our data suggest that neuronal dysregulation of energy homeostasis, potentially via disturbed leptin signaling, may contribute to the observed weight gain and altered insulin/glucose tolerance.

## 2. Results

### 2.1. Maternal BDE-47 Exposure Resulted in Sex-Specific Weight Gain and Impaired Insulin and Glucose Tolerance in the Offspring

**Figure 1:**
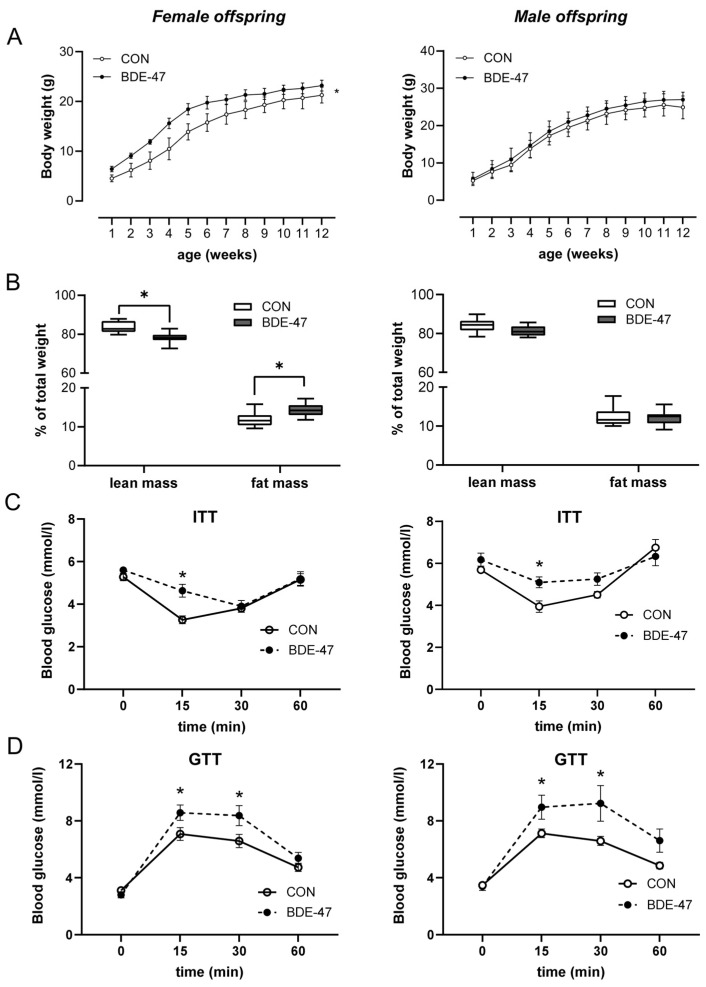
Maternal exposure to BDE-47 and weight development and glycose metabolism in the offspring. (**A**) Bodyweight development and (**B**) body composition are shown for female (**left**) and male offspring (**right**) from BDE-47-exposed dams, (**C**) insulin tolerance test (ITT), and (**D**) glucose tolerance test (GTT) were performed in 9-week- and 10-week-old offspring, respectively. Data are expressed as mean ± SEM, *n* ≥ 12. Significant differences were derived by two-way ANOVA (**A**) or Mann–Whitney test (**B**–**D**) with * *p* < 0.05.

**Figure 2:**
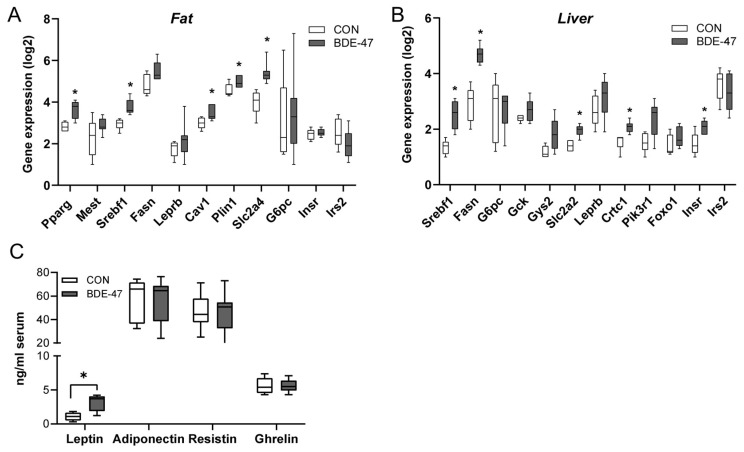
Maternal BDE-47 exposure and expression of key genes in adipose tissue and liver and serum adipokines in the female offspring. (**A**) The mRNA expression levels of selected target genes investigated in adipose tissue and (**B**) liver of 12-week-old female offspring. (**C**) Levels of leptin, adiponectin, resistin, and ghrelin measured in serum of female offspring. Data are expressed as mean ± SEM, *n* ≥ 5. Significant differences were derived by Mann–Whitney test with * *p* < 0.05.

**Figure 3:**
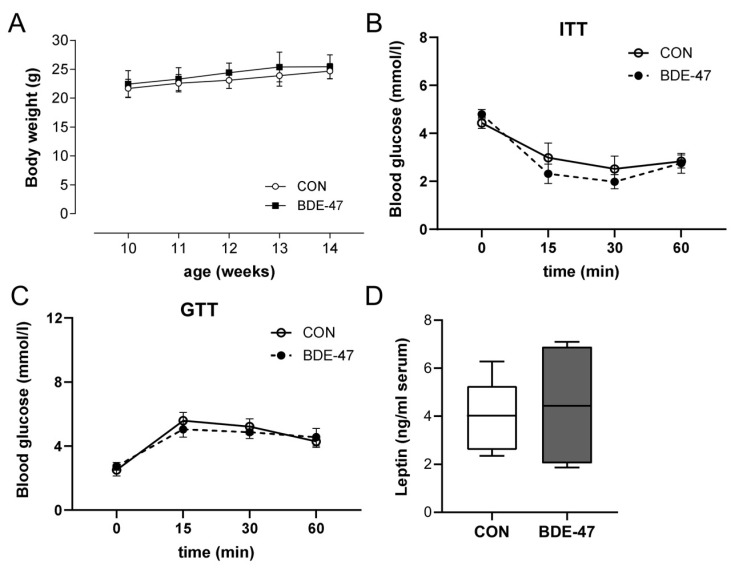
Effect of BDE-47 exposure to adult mice on weight development, glycose metabolism and leptin serum levels. (**A**) Bodyweight development, (**B**) ITT, (**C**) GTT and (**D**) leptin serum levels are shown from adult female mice exposed to BDE-47. Data are expressed as mean ± SEM, *n* ≥ 10.

Using a cross-generational mouse model (Supplementary Figure S1), female offspring from dams exposed to BDE-47 showed a significantly higher weight than control animals over the entire observation period (Figure 1A, left column). The increased body weight became noticeable shortly after birth and was associated with higher fat and lower lean mass, as measured by whole-body composition analysis using nuclear magnetic resonance technology (Figure 1B). In contrast, the weight of the male offspring of BDE-47-exposed dams was not significantly affected by maternal exposure to BDE-47 and no difference in lean muscle and fat mass was observed (Figure 1A,B, right column). However, both female and male offspring of dams exposed to BDE-47 developed an impaired insulin and glucose tolerance; showing significant differences in ITT and GTT compared to control mice (Figure 1C,D). Since the generationally mediated weight-increasing effect of BDE-47 was mainly observed in the female offspring, gene expression analyses were performed in adipose tissue of female mice at the end of the observation period of 12-weeks. The results revealed an increased mRNA expression of peroxisome proliferator-activated receptor gamma (ppar-γ), a transcription factor important for lipid uptake and adipo-genesis, caveolin-1 (cav-1), and perilipin-1 (plin-1), both of which regulate the metabolism of lipid droplets and glucose transporter 4 (glut4, slc2a4) in the offspring of BDE-47-exposed dams (Figure 2A). In addition, transcript of sterol regulatory element-binding transcription factor 1 (scrbf1), which plays a key role in lipogenesis, is augmented in adipose tissue and liver. In the liver, expression of insulin receptor 1 (insr-1) and glucose transporter 2 (slc2a2) was also increased compared to the offspring of control mice (Figure 2B). While serum leptin levels were significantly increased in the female offspring of BDE-47-exposed dams when compared to the controls, adiponectin, resistin, and ghrelin concentrations were not affected (Figure 2C). Quite remarkable is that a direct exposure of adult female mice to low-dose BDE-47 had no effects on weight, insulin, and glucose tolerance, or serum leptin levels (Figure 3A–C). These data demonstrate the importance of the developmental period as a particularly vulnerable time for chemical exposure, which affects disease development later in life.

### 2.2. Impact of BDE-47 Exposure on Murine and Human Adipocyte Development In Vitro

**Figure 4:**
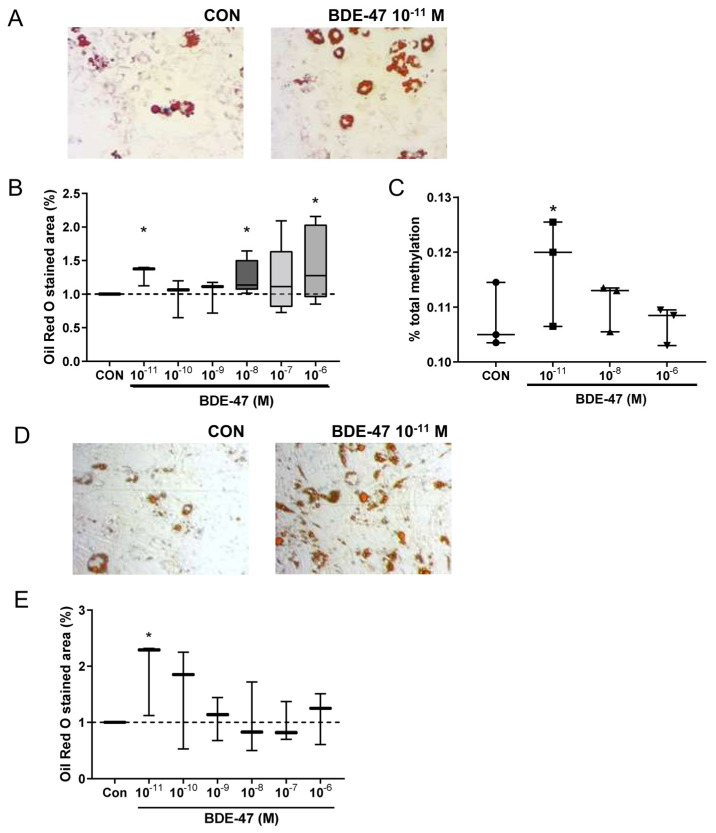
Effect of BDE-47 exposure on adipocyte differentiation. (**A**) Representative Oil Red O-stained pictures after differentiation of 3T3L1 mouse preadipocytes in the presence of BDE-47 (scale bar: 100 µm). (**B**) Triglyceride storage of mouse adipocytes assessed via Oil Red O staining. (**C**) Representative Oil Red O-stained pictures after differentiation from human MSCs in the presence of BDE-47 (**D**) Representative Oil Red O-stained pictures after adipocyte differentiation from MSC in the presence of BDE-47 (scale bar: 100 µm). (**E**) Triglyceride storage of human adipocytes assessed via Oil Red O staining. Data are expressed as mean ± SEM ofn = 3 experiments. Significant differences were derived by ANOVA with * *p* < 0.05.

To better understand the mechanistic pathways underlying the observed obesogenic effects, we next evaluated the potential effect of BDE-47 on adipocyte differentiation. For this, we employed 3T3-L1 mouse preadipocytes and treated them with different BDE-47 concentrations from 10 pM to 1 μM during culture. Interestingly, both the very low and the higher BDE concentrations showed an increased amount of triglyceride storage, while the medium concentrations had no effect (Figure 4A,B). These results indicate a U-shaped influence of BDE-47 on adipogenesis.

To evaluate a possible involvement of epigenetic modifications in mediating the adipogenic effect of BDE-47 a global DNA methylation assay based on detection and quantitation of 5-methylcytosine (5-mC) was used in the 3T3L1 cells. Here, BDE-47 treatment at a very low concentration (10 pM) induced an increased DNA methylation (Figure 4C).

In order to translate the murine results of BDE-47 in adipocyte differentiation to the situation in humans, an established differentiation assay for human mesenchymal stem cells (MSC) was applied [9]. Similar to the results observed for the murine cells, BDE-47 treatment already at low concentration of 10 pM increased adipocyte differentiation of human MSC, assessed by the amount of triglyceride storage (Figure 4D). These results underline the potential obesogenic causality of BDE-47 driven effects.

### 2.3. Maternal BDE-47 Exposure Induced a Disturbed Regulation of Energy Homeostasis in Female Offspring

**Figure 5:**
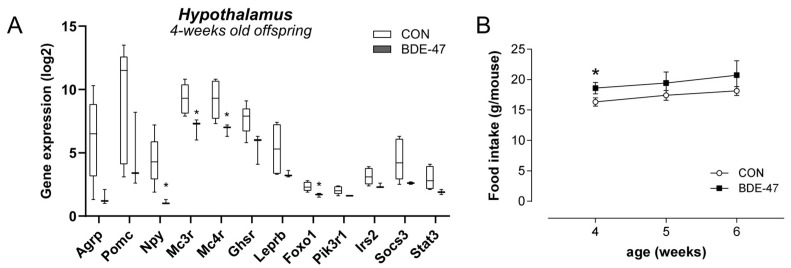
Maternal BDE-47 exposure and expression of key genes in hypothalamus and food intake of 4-week-old female offspring. (**A**) The mRNA expression levels of selected target genes investigated in hypothalamus of 4-week-old female offspring. (**B**) Food intake of 4–6-week-old female offspring from BDE-47-exposed dams compared to control mice. Data are expressed as mean ± SEM, *n* ≥ 5. Significant differences were derived by Mann–Whitney test with * *p* < 0.05.

To additionally investigate whether BDE-47 exposure during the prenatal and lactational period also affects the central regulation of satiety and hunger in the offspring immediately after weaning, an analysis of the genes involved in this regulation in the hypothalamus was carried out. Data show that melanocortin type 3 and 4 receptor (mc3r, mc4r) expression was significantly downregulated in 4-week old offspring from BDE-47-exposed dams, as was forkhead transcription factor-1 (foxo1) and neuropeptide Y (npy, Figure 5A). Other genes, such as agouti-related neuropeptide gene (agrp), pro-opiomelanocortin (pomc), insulin receptor substrate 2 (irs2), and the leptin receptor (lepr) also seemed to have a reduced expression, but this was not significant. Investigating the feeding behaviour directly after weaning revealed a higher food intake in the offspring from BDE-47-exposed dams compared to control mice from unexposed dams (Figure 5B). It should be noted that gene expression in fat and liver remained unaffected in the offspring of BDE-47-exposed dams directly after weaning (Supplementary Figure S3A,B). These results suggest that early weight gain is due to impaired central satiety regulation, followed by increased adipogenesis manifesting at a later stage.

### 2.4. Offspring Weight Gain Caused by Maternal BDE-47 Exposure Is Associated with DNA Hypermethylation

**Figure 6:**
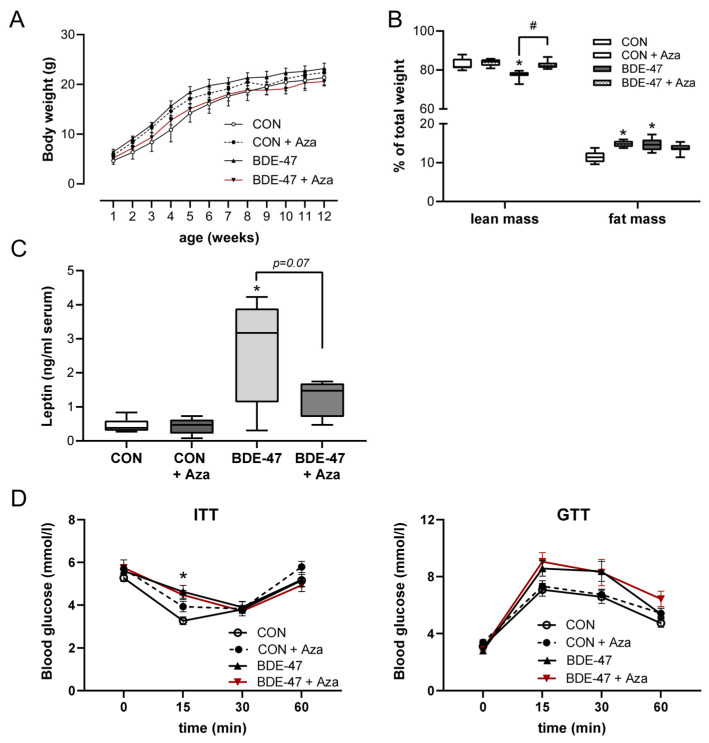
Offspring weight gain caused by maternal BDE-47 exposure is reversed by treatment with a DNA methyltransferase inhibitor. After treatment of F1 mice with the DNA methyltransferase inhibitor Aza, weight development (**A**), body composition (**B**), leptin serum levels (**C**), and ITT and GTT (**D**) were evaluated. Data are expressed as mean ± SEM, *n* ≥ 7. (Significant differences were derived by two-way ANOVA or Mann–Whitney test with * *p* < 0.05, BDE-47 or CON + Aza vs. CON, ^#^ *p* < 0.05, BDE-47 vs. BDE-47 + Aza.

The results obtained with BDE-47 in the in vitro differentiation of adipocytes suggest the involvement of DNA hypermethylation. To assess whether this also applies in vivo with regard to maternal BDE-47 exposure and the development of overweight in the offspring, one-week-old pups from BDE-47-exposed dams were treated with the DNA methyltransferase inhibitor 5-aza-2′-deoxycytidine (Aza) for two weeks until weaning [9,19]. Treatment of the offspring with Aza reduced the body weight and increased the lean mass (Figure 6A,B). Although Aza treatment had an increasing effect on fat mass in control animals, Aza also reduced fat mass in the offspring of BDE-47-exposed dams, but not significantly (Figure 6B). Leptin levels after Aza treatment were diminished but without reaching significance (Figure 6C). Furthermore, it is very interesting that Aza treatment had no effect on the altered insulin and glucose tolerance in the offspring of dams exposed to BDE-47 (Figure 6D). These results suggest an important role of the DNA hypermethylation in the observed BDE-47-induced weight gain, but not in the altered insulin and glucose tolerance.

## 3. Discussion

This study applied a cross-generational mouse model as well as murine und human in vitro adipocyte differentiation assays to investigate the effects of maternal exposure to the flame-retardant BDE-47 on offspring weight development. In vivo, maternal exposure of mice to BDE-47 resulted in weight gain in the female offspring together with an altered glucose and insulin tolerance. Impaired glucose and insulin tolerance was also observed in males; however, this was not accompanied by weight gain. Furthermore, it is difficult to assess the extent to which delayed glucose metabolism has a physiological relevance. The observed weight gain appears to be due to increased adipogenesis induced by BDE-47, additionally driven via an impaired satiety regulation in the hypothalamus of the offspring.

Since there is evidence from some epidemiological studies that maternal PBDE exposure may be associated with higher BMI in children [15] and may also lead to impaired energy metabolism and an increased risk of type 2 diabetes [16], it is particularly important to verify and deepen these results using in vivo disease models to proof a causal relationship between exposure and disease. To do so, the use of relevant exposure concentrations in the experimental model is a crucial point. The dose of BDE-47, the most abundant PBDE in both the environment and human bodies [20], was determined from an acceptable daily human intake of 0.02 µg kg^−1^ body weight based on no observed adverse effect levels (NOAELs) (0.002 mg kg^−1^ body weight/day) reported in intrauterine exposure studies [17,21]. In previous work, where we examined the health effects of other EDCs, such as phthalates or parabens, we found that exposure to ADI concentrations in mice resulted in comparable serum concentrations to those measured in highly exposed mothers included in our prospective mother–child cohort study LINA [9,19]. Notably, the used BDE-47 dose had no direct impact on weight or other metabolic parameters in adult female mice, confirming that only exposure during the critical period of pregnancy and lactation resulted in weight gain in offspring. This highlights the importance of developmental exposures in disease risk later in life. Interestingly, the effect on weight was sex-specific, as it was only observed in females. This is in contrast to another experimental study investigating the effect of perigestational BDE-47 exposure in Sprague–Dawley rats. Here, the authors describe a weight gain in male but not in female offspring [18]. However, the BDE-47 concentration in that approach was many times higher than the one used in the present study. Different concentrations of hormonally active chemicals can probably not only lead to different sex-specific effects but can also be mediated via other mechanisms. This is particularly interesting because effects of EDCs often follow non-monotonic dose–response, such as a U-shaped curve [22,23]. We also obtained such a u-shaped curve in our in vitro experiments in which broad ranges of different concentrations were used during murine adipocyte differentiation. Both very low concentrations (10 pM) and higher concentrations (1 µM) showed an impact on triglyceride storage, while at the intermediate concentrations there was no effect. While BDE-47 has previously been shown to promote adipocyte differentiation at higher concentrations [24,25], an effect of ultra-low concentrations, as presented in the current project, has not been documented before. Furthermore, we were even able to confirm an adipogenic effect of low-dose BDE-47 in both mice and humans, which suggests that our results could indeed be of general (patho)physiological relevance. Another interesting finding is that altered DNA methylation was only observed at the low BDE-47 concentration, although higher concentrations had a similar effect on triglyceride storage. This could suggest different underlying mechanisms of the concentration ranges applied. An epigenetic regulation of adipogenesis has already been described in previous work [26], as has modulation of DNA methylation by BDE-47 [27].

The increased triglyceride storage induced by BDE-47, as we observed during adipocyte differentiation, is consistent with the increased expression of transcripts in adipose tissue of the female offspring of BDE-47-exposed dams involved in adipogenesis and lipid metabolism (Pparγ, Cav-1, Plin-1, Scrbf1). In addition, there is enhanced expression of glucose transporters in adipose tissue (Slc2a4) and liver (Slc2a2), which may be a (so far unsuccessful) compensating response to the elevated blood glucose levels: glucose levels in the offspring of BDE-47-exposed dams are elevated in both ITT and GTT compared to control animals, with no detected differences in basal glucose levels. These findings indicate an impaired insulin sensitivity that may point to an early development of type 2 diabetes. In fact, there are initial indications from epidemiological studies that exposure to BDE-47 [28] or other PBDEs [16] may be associated with increased diabetes prevalence. In contrast to the weight increase in the female offspring of BDE-47-exposed dams, the impaired insulin sensitivity in GTT and ITT affects both sexes. Why this imbalance in the glucose/insulin metabolism is not associated with weight gain in the male offspring requires further research but suggests different initial molecular events of BDE-47, in terms of weight and metabolism, and reinforces the need to include both biological sexes in risk assessment of chemicals. As expected, serum leptin levels are only increased in the weight-/fat-accumulating female offspring. However, the expected satiety signal of leptin via the following signal transduction in the hypothalamus obviously was not sufficient due to the higher food intake already shown of the young female offspring. Potentially a leptin resistance could contribute to the impaired weight gain and insulin sensitivity, as such an association has been previously considered [29]. This hypothesis was supported by our mRNA expression analyses of the hypothalamus of 4-week-old female offspring of mothers exposed to BDE-47. Here we found significantly reduced expression of Mc3r and Mc4r compared to control mice. Both receptors are indeed linked with leptin resistance [30]. In general, the leptin-associated central regulation of satiety and hunger appears to be affected by BDE-47 with a downregulation of orexigenic genes such as Foxo1 or Npy. Foxo1 was shown to be involved in the hypothalamic control of energy homeostasis, as its ablation in hypothalamic Pomc neurons reduced food intake and body weight. Furthermore, Foxo1 was shown to interfere with both adipogenesis and glucose/insulin metabolism [31]. However, although Foxo1 was downregulated, exposure to BDE-47 was rather associated with an increased weight and fat mass, together with an early increased food intake, underlining the disturbance of the central regulation in this context. It should be noted that all RNA expression data shown in the current study still needs to be verified at the protein level. However, due to the limited organic material available per animal (particularly for the hypothalamus) we chose gene expression analyses for broader hypothesis generation rather than confirmatory protein levels when conceptualizing the experimental setup and the analytical protocol. Therefore, our interpretation remains hypothesis-driven and partly speculative. We must also acknowledge the small sample size as a limitation for the hypothalamic data as well as the lack of analysis of specific regions of the hypothalamus. Further experiments and analysis are required to better evaluate the hypothesis proposed in the present study. Nevertheless, the reduced expression of genes in the hypothalamus in our study is consistent with gene expression data from female offspring of dams exposed to a PBDE mixture that also contained BDE-47 [32]. In line, the authors saw a downregulation of genes such as Agrp, NPY, or Mc4r. However, they did not observe any weight gain in this study [32]. This could be due to an interaction of the different PBDEs in the mixture.

Early life exposure and the above-mentioned gene expression changes might be mediated via epigenetic modifications [9]. Although several studies have rather shown a BDE-47-induced hypomethylation [25,27], results from our in vitro differentiation of mouse adipocytes as well as results from other studies [33,34], suggest the involvement of an increased DNA methylation in mediating the BDE-47-induced effects. Interestingly, hypermethylation was only observed at very low BDE-47 concentrations, suggesting different mechanisms that led to increased adipocyte differentiation at low or at higher concentrations. In the present analyses, treating pups with the DNA methyltransferase inhibitor Aza reduced the BDE-47-induced weight gain and leptin serum levels and increased the lean mass. However, it is noteworthy that Aza-induced hypomethylation alone resulted in increased weight and fat mass in unexposed control animals. This could be because hypomethylation can lead to increased expression of genes involved in adipogenesis, such as PPARg [25]. Based on that observation, it is even more surprising that Aza can still reverse the BDE-47-driven effect on weight development, probably by increasing the expression of anti-adipogenic genes, which still have to be identified. It is also interesting that Aza has no effect on impaired insulin sensitivity in the offspring of BDE-47-exposed dams. As indicated above, there seem to be different underlying mechanisms/molecular initial events of BDE-47 in terms of weight and metabolism. Anyhow, further investigations are needed to clarify the epigenetic alterations more specifically. We also cannot exclude that additional epigenetic regulations, like histone modifications, as shown, e.g., for Mc4r [35] or other pathways, might be involved in mediating the BDE-47-induced changes in weight development and energy homeostasis.

In summary, our study results show that maternal exposure to very low concentrations of BDE-47 results in overweight development in the female offspring. The effect is mediated by an enhanced adipogenesis and might be promoted by a disturbed neuronal regulation of food intake. Perinatal BDE-47 exposure also led to an impaired insulin sensitivity in both sexes that might be associated with an observed disturbed leptin signaling. Our results draw attention to the great importance of exposure to EDCs, even at very low concentrations, during pregnancy and breastfeeding, for disease susceptibility in later life.

## 4. Materials and Methods

### 4.1. Mice

Balb/cByJ mice (6–8 weeks of age) were purchased from the Elevage Janvier Laboratory (Le Genest St Isle, France) with a 7-day adaption period before the start of experiments. Animals were maintained in groups of 3–6 mice per cage in the animal facility at the University of Leipzig (Germany) under conventional conditions with 21.5–23 °C room temperature, an average of 55% humidity, and a 12-h day/night rhythm. Exposed and control dams as well as the offspring of exposed and control mice were housed separately. All mice were kept in multiple sealed cages with HEPA filters by Sealsafe^®^ and bedded with LIGNOCEL^®^ bedding material. Dams and pups received a phytoestrogen-free diet (C1077 from Altromin, Lage, Germany) and water ad libitum. All animal experiments were performed at least 2 times with at least 3 dams per group with a maximum of 4 pups per sex per dam. All animal experiments were conducted in accordance with institutional and state guidelines. Animal protocols used in this study were approved by the Committee on Animal Welfare of Saxony/Leipzig (Permit Number: TVV01/15, 14/18).

### 4.2. Low-Dose Exposure to BDE-47

To evaluate the impact of an exposure during the prenatal and lactational period on weight development in the offspring we exposed female Balb/c mice to BDE-47 (0.02 µg/kg body weight/day) orally administered by gavage in 200 μL corn oil twice per week. The intervention lasted from one week before mating with BALB/c males until weaning of the pups at 4 weeks. Female mice received a BDE-47 concentration estimated from a calculated human acceptable intake based on earlier intrauterine exposure studies [21]. Control dams received the vehicle by gavage. To investigate a possible involvement of epigenetic alterations, offspring were treated i.p. with Aza (160 µg/kg body weight, Sigma-Aldrich, Munich, Germany) dissolved in PBS 3 times per week starting 1 week after birth until weaning [9,19]. Food intake was monitored after weaning until week 6.

### 4.3. Weight Assessment, Insulin and Glucose Tolerance Test and Metabolic Serum Proteins

Body weight of the pups was measured twice a week, and a mean weight per week was calculated for each mouse. At the end of the observation period (12 weeks), whole-body composition (fat mass and lean mass) was determined in awake mice based on nuclear magnetic resonance technology using an EchoMRI700™ instrument (Echo Medical Systems, Houston, TX, USA) in the offspring of control and BDE-47 exposed dams. An insulin tolerance test (ITT) was performed in the offspring 9 weeks after birth. Insulin (0.75 U/kg body weight) was injected intraperitoneally (i.p.). For glucose measurements blood from the tail vein was taken at four time points at 0, 15, 30 and 60 min after insulin injection. For the glucose tolerance test (GTT), glucose (2 g/kg body weight) was injected i.p. into fasting mice (10 weeks after birth), and the glucose measurement was performed equally to ITT. Adiponectin, leptin, resistin, and acetylated ghrelin serum concentrations were determined by ELISA using mouse standards according to the manufacturer’s guidelines (mouse adiponectin, leptin, resistin ELISA; R&D Systems, Minneapolis, MN, USA), (mouse/rat acylated ghrelin ELISA; BioVendor, Karasek, Czech Republic).

### 4.4. In Vitro Adipocyte Differentiation

For adipocyte differentiation, 3T3L1 mouse preadipocytes were used according to Ruiz-Ojeda et al. [35]. In brief, for differentiation, 3T3-L1 cells were passaged onto 12-well plates at a concentration of 6000 cells/cm2 and were allowed to confluent for 48 h in basal media (BM; 90% DMEM, 10% FBS, 1% Ala/Gln, 0.1% Pen/Strep). After another 48 h in BM, cells were placed into differentiation medium (BM + 1 µM dexamethasone, 0.5 mM IBMX, 1 µg/mL Insulin) along with 50 µL of exposure solution (BDE-47: 10^−11^–10^−6^ M in maximal 0.05% ethanol or respective solvent control) for another 48 h. Finally, they were placed in maintenance medium (BM + 1 µg/mL Insulin) with the same 50 µL of exposure solution. The medium was changed every 2–3 days for another 8 days until they were harvested and processed for further analyses. For Oil Red O staining, cells were stained with Oil Red O for triglyceride depots and quantified via absorbance measurement at 510 nm.

Human adipose-derived mesenchymal stem cells derived from a female donor (MSC; ATCC^®^, PCS-500-011; #59753760) and appropriate culture media were purchased from LGC Standards (Wesel, Germany). Cells were cultured under standardized conditions at 37 °C, 5% CO_2_, and 95% humidity according to the manufacturer’s instructions, as described previously [10]. In brief, for adipocyte differentiation, MSC passages 3–5 were seeded at 9.000 cells/cm^2^ in a 96-well plate and grown to 70% confluence. For initiation of differentiation, cells were fed with adipocyte differentiation initiation medium (ADIM; ATCC Adipocyte Differentiation Toolkit PCS-500-050). ADIM was changed to adipocyte maintenance medium (ADMM; ATCC^®^ Adipocyte Differentiation Toolkit PCS-500-050) after 4 days and changed regularly. Cells were treated with BDE-47 at different concentrations during the entire differentiation period. After a total of 16 days, cells were stained with Oil Red O for triglyceride depots.

### 4.5. RNA Extraction, cDNA Synthesis, and qPCR

Dissection of the hypothalamus was conducted from the ventral side of the brain. The optic chiasm was removed away from the anterior portion of the hypothalamus. The mammillary nuclei were dissected from the posterior of the hypothalamus. The entire hypothalamus was prepared, including the arcuate, ventromedial, dorsomedial, and paraventricular nuclei. Total RNA was extracted from adipocytes of humans, 3T3-L1 cells, visceral adipose tissue, liver, and hypothalamus of mice by using QIAzol Lysis Reagent (QIAGEN, Hilden, Germany) and RNAeasy Plus Mini Kit (QIAGEN) following the manufacture’s instructions; 200 ng were used for cDNA synthesis by RevertAid™ H Minus Reverse Transcriptase (Thermo Fisher Scientific, Waltham, MA, USA). Primers (Supplementary Table S1) were designed using the web-based Primer3Plus package (www.primer3plus.com assessed on 3 April 2024). The semi-quantitative PCR was performed on the Biomark HD system (Standard BioTools, San Francisco, CA, USA) using EvaGreen DNA binding dye with BioMark™ 48.48 Dynamic Array Integrated Fluidic Circuits according to the manufacture’s recommendations. Gene expression was determined via the 2^∆∆CT^ method [36,37]. Data were normalized to geometric mean of the four reference genes ATP synthase subunit b (*atp5f1*), eukaryotic elongation factor 2 (*eef2*), retention in endoplasmic reticulum sorting receptor 1 (*rer1*), and ribosomal protein l13a (*rpl13a*). Stability of reference genes was checked with the geNorm algorithm implemented in the open-source qbase+ data analysis software (www.qbaseplus.com assessed on 6 May 2024).

### 4.6. Statistical Analysis

Experimental data sets from in vivo mouse studies and in vitro experiments were processed and analyzed in GraphPad PRISM 7.02 for Windows (GraphPad Software, Inc., Boston, MA, USA). Data were expressed as mean ± SEM or min to max, and *p* values of less than 0.05 were considered significant by Wilcoxon–Mann–Whitney test or ANOVA.

## Data Availability

The data generated during this study is available upon request.

## Supplementary Materials

The following supporting information can be downloaded at: https://www.mdpi.com/article/10.3390/ijms25168620/s1.

## References

[1] Ng M., Fleming T., Robinson M., Thomson B., Graetz N., Margono C., Mullany E.C., Biryukov S., Abbafati C., Abera S.F. (2014). Global, regional, and national prevalence of overweight and obesity in children and adults during 1980–2013: A systematic analysis for the Global Burden of Disease Study 2013. Lancet.

[2] OECD (2017). OECD Obesity Update. www.oecd.org/els/health-systems/Obesity-Update-2017.pdf.

[3] Heindel J.J., vom Saal F.S. (2009). Role of nutrition and environmental endocrine disrupting chemicals during the perinatal period on the aetiology of obesity. Mol. Cell. Endocrinol..

[4] Heindel J.J., Vom Saal F.S., Blumberg B., Bovolin P., Calamandrei G., Ceresini G., Cohn B.A., Fabbri E., Gioiosa L., Kassotis C. (2015). Parma consensus statement on metabolic disruptors. Environ. Health A Glob. Access Sci. Source.

[5] Gillman M.W., Ludwig D.S. (2013). How early should obesity prevention start?. N. Engl. J. Med..

[6] Rubin B.S., Soto A.M. (2009). Bisphenol A: Perinatal exposure and body weight. Mol. Cell. Endocrinol..

[7] Janesick A., Blumberg B. (2012). Obesogens, stem cells and the developmental programming of obesity. Int. J. Androl..

[8] Martino D., Prescott S. (2011). Epigenetics and prenatal influences on asthma and allergic airways disease. Chest.

[9] Leppert B., Strunz S., Seiwert B., Schlittenbauer L., Schlichting R., Pfeiffer C., Roder S., Bauer M., Borte M., Stangl G.I. (2020). Maternal paraben exposure triggers childhood overweight development. Nat. Commun..

[10] Junge K.M., Leppert B., Jahreis S., Wissenbach D.K., Feltens R., Grutzmann K., Thurmann L., Bauer T., Ishaque N., Schick M. (2018). MEST mediates the impact of prenatal bisphenol A exposure on long-term body weight development. Clin. Epigenetics.

[11] Dodson R.E., Nishioka M., Standley L.J., Perovich L.J., Brody J.G., Rudel R.A. (2012). Endocrine disruptors and asthma-associated chemicals in consumer products. Environ. Health Perspect..

[12] Bond G.G., Dietrich D.R. (2017). Human cost burden of exposure to endocrine disrupting chemicals. A critical review. Arch. Toxicol..

[13] Malliari E., Kalantzi O.I. (2017). Children’s exposure to brominated flame retardants in indoor environments—A review. Environ. Int..

[14] Li J., Zhao L., Letcher R.J., Zhang Y., Jian K., Zhang J., Su G. (2019). A review on organophosphate Ester (OPE) flame retardants and plasticizers in foodstuffs: Levels, distribution, human dietary exposure, and future directions. Environ. Int..

[15] Erkin-Cakmak A., Harley K.G., Chevrier J., Bradman A., Kogut K., Huen K., Eskenazi B. (2015). In utero and childhood polybrominated diphenyl ether exposures and body mass at age 7 years: The CHAMACOS study. Environ. Health Perspect..

[16] Lim J.S., Lee D.H., Jacobs D.R. (2008). Association of brominated flame retardants with diabetes and metabolic syndrome in the U.S. population, 2003–2004. Diabetes Care.

[17] Suvorov A., Battista M.C., Takser L. (2009). Perinatal exposure to low-dose 2,2′,4,4′-tetrabromodiphenyl ether affects growth in rat offspring: What is the role of IGF-1?. Toxicology.

[18] Gao H., Li P., Liu L., Yang K., Xiao B., Zhou G., Tian Z., Luo C., Xia T., Dong L. (2019). Perigestational low-dose BDE-47 exposure alters maternal serum metabolome and results in sex-specific weight gain in adult offspring. Chemosphere.

[19] Jahreis S., Trump S., Bauer M., Bauer T., Thurmann L., Feltens R., Wang Q., Gu L., Grutzmann K., Roder S. (2018). Maternal phthalate exposure promotes allergic airway inflammation over 2 generations through epigenetic modifications. J. Allergy Clin. Immunol..

[20] Birnbaum L.S., Staskal D.F. (2004). Brominated flame retardants: Cause for concern?. Environ. Health Perspect..

[21] Schrenk D., Bignami M., Bodin L., Chipman J.K., del Mazo J., Grasl-Kraupp B., Hogstrand C., Hoogenboom L.R., Leblanc J.-C., Nebbia C.S. (2024). Update of the risk assessment of polybrominated diphenyl ethers (PBDEs) in food. EFSA J..

[22] Hill C.E., Myers J.P., Vandenberg L.N. (2018). Nonmonotonic Dose-Response Curves Occur in Dose Ranges That Are Relevant to Regulatory Decision-Making. Dose Response.

[23] Vandenberg L.N. (2014). Non-monotonic dose responses in studies of endocrine disrupting chemicals: Bisphenol a as a case study. Dose Response.

[24] Yang C., Wong C.M., Wei J., Chung A.C.K., Cai Z. (2018). The brominated flame retardant BDE 47 upregulates purine metabolism and mitochondrial respiration to promote adipocyte differentiation. Sci. Total Environ..

[25] Kamstra J.H., Hruba E., Blumberg B., Janesick A., Mandrup S., Hamers T., Legler J. (2014). Transcriptional and epigenetic mechanisms underlying enhanced in vitro adipocyte differentiation by the brominated flame retardant BDE-47. Environ. Sci. Technol..

[26] Pant R., Firmal P., Shah V.K., Alam A., Chattopadhyay S. (2020). Epigenetic Regulation of Adipogenesis in Development of Metabolic Syndrome. Front. Cell Dev. Biol..

[27] Byun H.M., Benachour N., Zalko D., Frisardi M.C., Colicino E., Takser L., Baccarelli A.A. (2015). Epigenetic effects of low perinatal doses of flame retardant BDE-47 on mitochondrial and nuclear genes in rat offspring. Toxicology.

[28] Zhang Z., Li S., Liu L., Wang L., Xiao X., Sun Z., Wang X., Wang C., Wang M., Li L. (2016). Environmental exposure to BDE47 is associated with increased diabetes prevalence: Evidence from community-based case-control studies and an animal experiment. Sci. Rep..

[29] Amitani M., Asakawa A., Amitani H., Inui A. (2013). The role of leptin in the control of insulin-glucose axis. Front. Neurosci..

[30] Morris D.L., Rui L. (2009). Recent advances in understanding leptin signaling and leptin resistance. Am. J. Physiol. Endocrinol. Metab..

[31] Doan K.V., Kinyua A.W., Yang D.J., Ko C.M., Moh S.H., Shong K.E., Kim H., Park S.K., Kim D.H., Kim I. (2016). FoxO1 in dopaminergic neurons regulates energy homeostasis and targets tyrosine hydroxylase. Nat. Commun..

[32] Kozlova E.V., Denys M.E., Benedum J., Valdez M.C., Enriquez D., Bishay A.E., Chinthirla B.D., Truong E., Krum J.M., DiPatrizio N.V. (2022). Developmental exposure to indoor flame retardants and hypothalamic molecular signatures: Sex-dependent reprogramming of lipid homeostasis. Front. Endocrinol..

[33] Robinson J.F., Kapidzic M., Hamilton E.G., Chen H., Puckett K.W., Zhou Y., Ona K., Parry E., Wang Y., Park J.S. (2019). Genomic Profiling of BDE-47 Effects on Human Placental Cytotrophoblasts. Toxicol. Sci..

[34] Chen H., Seifikar H., Larocque N., Kim Y., Khatib I., Fernandez C.J., Abello N., Robinson J.F. (2019). Using a Multi-Stage hESC Model to Characterize BDE-47 Toxicity during Neurogenesis. Toxicol. Sci..

[35] Tabachnik T., Kisliouk T., Marco A., Meiri N., Weller A. (2017). Thyroid Hormone-Dependent Epigenetic Regulation of Melanocortin 4 Receptor Levels in Female Offspring of Obese Rats. Endocrinology.

[36] Ruiz-Ojeda F.J., Ruperez A.I., Gomez-Llorente C., Gil A., Aguilera C.M. (2016). Cell Models and Their Application for Studying Adipogenic Differentiation in Relation to Obesity: A Review. Int. J. Mol. Sci..

[37] Livak K.J., Schmittgen T.D. (2001). Analysis of relative gene expression data using real-time quantitative PCR and the 2(-Delta Delta C(T)) Method. Methods.

