# Supplementary material for: Maternal Exposure to Low-Dose BDE-47 Induced Weight Gain and Impaired Insulin Sensitivity in the Offspring
Source: Int J Mol Sci. 2024 Aug 7;25(16):8620. doi: 10.3390/ijms25168620 (PMC11354368; doi:10.3390/ijms25168620)
Supplement: Supplementary file 1 [file ijms-25-08620-s001.zip › ijms-3090296-supplementary Table S1.pdf]

Supplementary Table S1. Primer for gene expression analysis.

Gene	Forward primer	Reverse primer
Agrp	GCCTCAAGAAGACAACTGCAGAC	AAGCAGGACTCGTGCAGCCTTA
Atp5f1	GTCCAGGGGTATTACAGGCAA	TCAGGAATCAGCCCAAGACG
Cav1	CACACCAAGGAGATTGACCTGG	CCTTCCAGATGCCGTCGAAACT
Crtc1	CGTGAACCAGATTGGAAGCAGC	CAAGTCTGCCACGCTCACGATA
Eef2	CAGAAGTACCGTTGTGAGCTGC	GTCAGAGGTTGGCACCATCTTG
Fasn	CACAGTGCTCAAAGGACATGCC	CACCAGGTGTAGTGCCTTCCTC
Foxo1	AGTGGATGGTGAAGAGCGTG	TTCTCCGGGGTGATTTTCCG
G6pc	AGGTCGTGGCTGGAGTCTTGTC	GTAGCAGGTAGAATCCAAGCGC
Gck	ACTTCAGGGTGATGCTGGTG	GGAAGGAGAAGGTGAAGCCC
Ghsr	AGTCTCATCGGGAGGAAGCT	ACAAAGGACACCAGGTTGCA
Gys2	CAGCACCATTAGACGAATCGGAC	CCAAGGTGACAACCTCGGACAA
Insr	GATGTCCCATCAAATATTGCCAAA	CGGCACGTACACAGAAGATG
Irs2	CACAACCTATCGTGGCACCT	AAGGTCTCTGAACTGTGGCG
Lepr	AACTGCAGTCTTCGGGGATG	GCTGCAGTGACATCAGAGGT
Mc3r	GGCCACTGCAAGGTAGATGT	GAGAACAAGGAGGCAGACCC
Mc4r	TATGCCCTCCGGAGTCAAGA	ATCGGGCCAGAGTGACAAAG
Mest	CCGGCTCACCATAAAGAGTC	ACGGTCCAAAGACTGGAGTG
Npy	TACTCCGCTCTGCGACACTACA	GGCGTTTTCTGTGCTTTCCTTCA
Pik3r1	CAGTTTGCCCCTCCTGATGTT	CGTGTACGTCGATCATCTCCA
Plin1	CTCTGGGAAGCATCGAGAAG	GATCCACATGGCCAGAGAGT
Pomc	AGAGACTAGGCCTGACACGT	CGTACTTCCGGGGGTTTTCA
Pparg	AAGAGCTGACCCAATGGTTG	GCATCCTTCACAAGCATGAA
Rer1	GCCTTGGGAATTTACCACCT	CTTCGAATGAAGGGACGAAA
Rpl13a	AGCCTACCAGAAAGTTTGCTTAC	GCTTCTTCTTCCGATAGTGCATC
Slc2a2	GTTGGAAGAGGAAGTCAGGGCA	ATCACGGAGACCTTCTGCTCAG
Slc2a4	GGTGTGGTCAATACGGTCTTCAC	AGCAGAGCCACGGTCATCAAGA
Socs3	CACACAAGGAGCCAAACACA	TAGCCACCTGGGTGAATCC
Srebf1	CGACTACATCCGCTTCTTGCAG	CCTCCATAGACACATCTGTGCC
Stat3	GTTCCTGGCACCTTGGATT	CAACGTGGCATGTGACTCTT
