# Supplementary material for: Maternal Exposure to Low-Dose BDE-47 Induced Weight Gain and Impaired Insulin Sensitivity in the Offspring
Source: Int J Mol Sci. 2024 Aug 7;25(16):8620. doi: 10.3390/ijms25168620 (PMC11354368; doi:10.3390/ijms25168620)
Supplement: Supplementary file 1 [file ijms-25-08620-s001.zip › ijms-3090296-supplementary.pptx]

## Slide 1
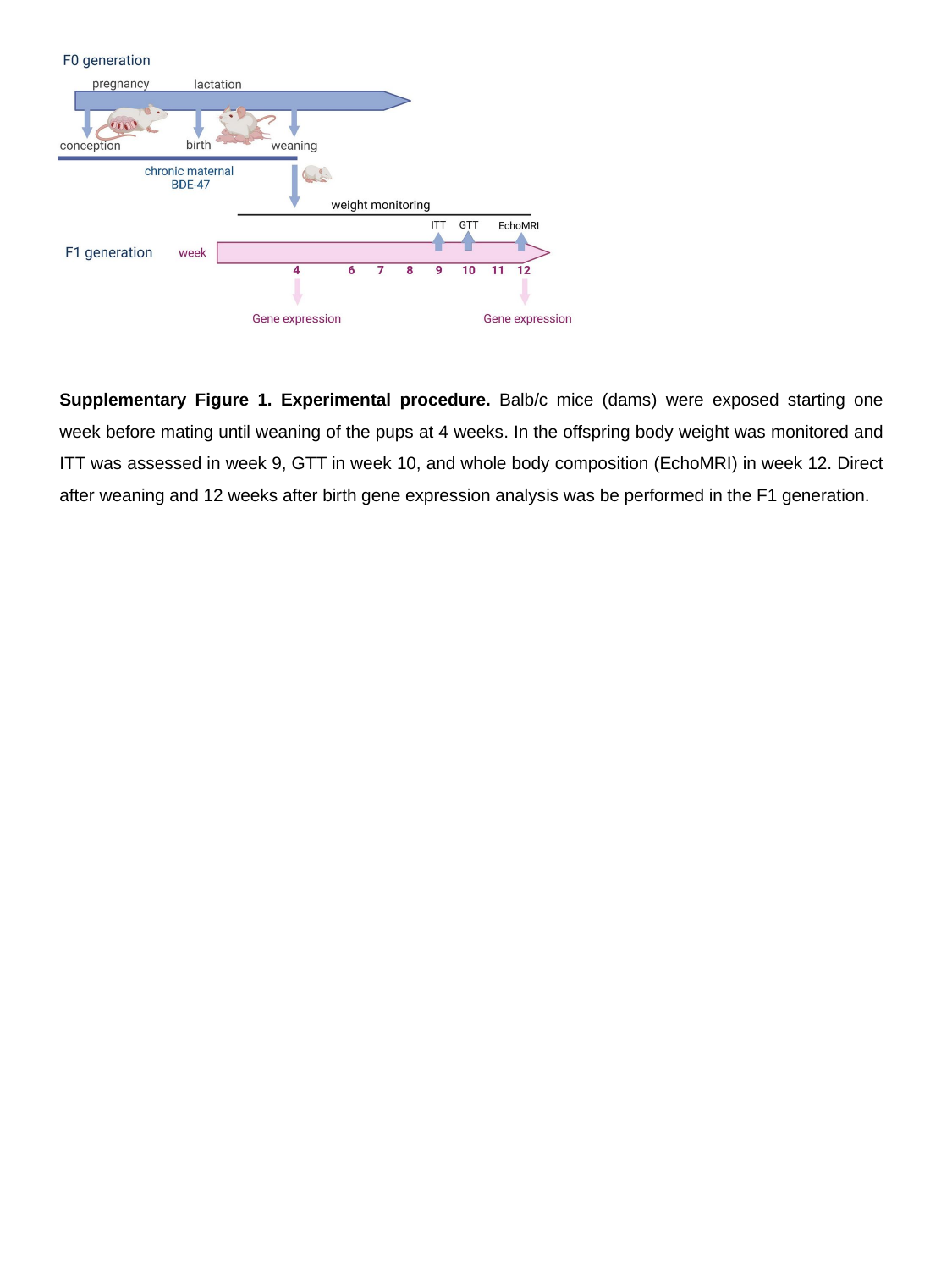

Supplementary Figure 1. Experimental procedure. Balb/c mice (dams) were exposed starting one week before mating until weaning of the pups at 4 weeks. In the offspring body weight was monitored and ITT was assessed in week 9, GTT in week 10, and whole body composition (EchoMRI) in week 12. Direct after weaning and 12 weeks after birth gene expression analysis was be performed in the F1 generation.

## Slide 2
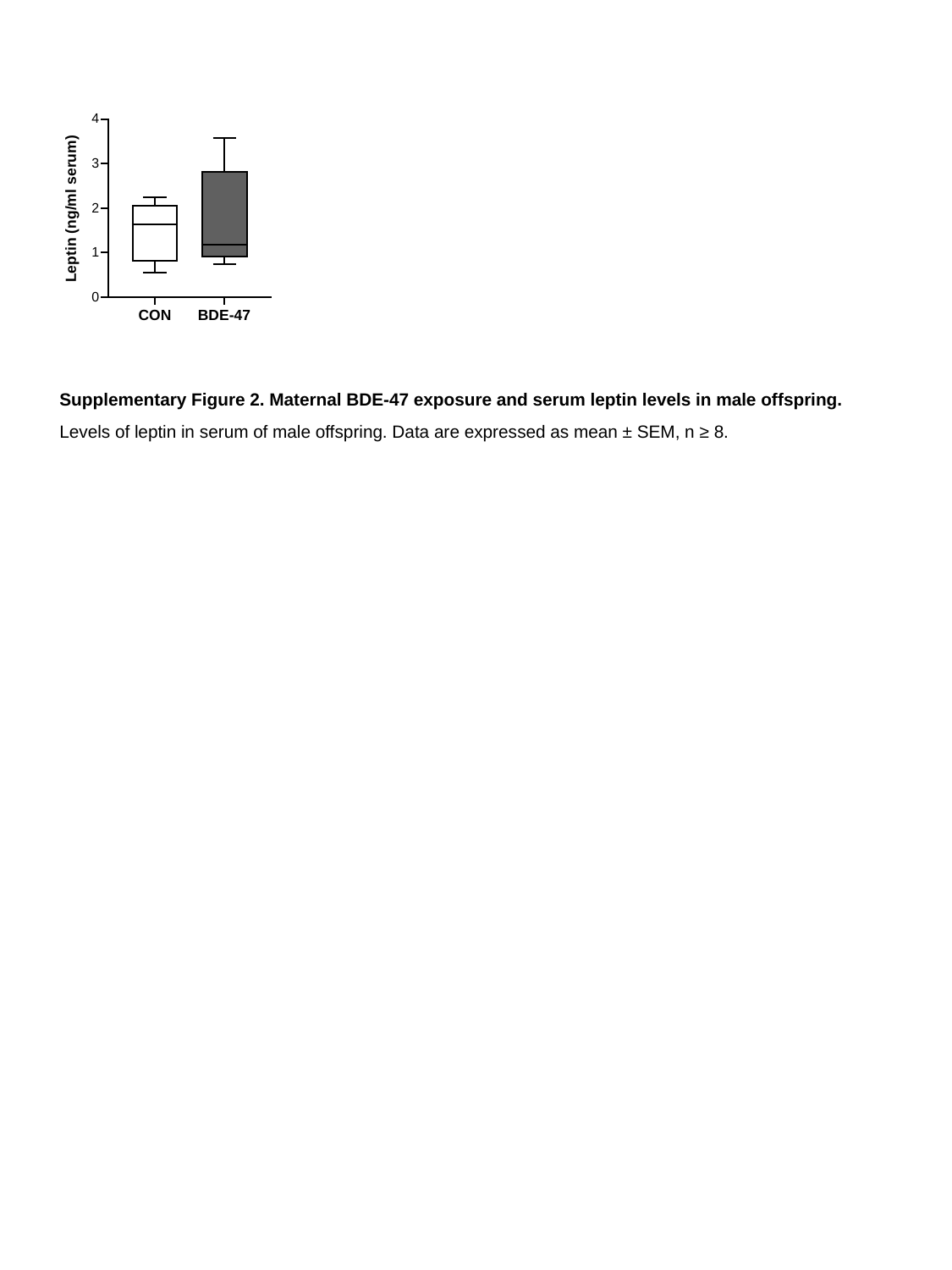

Supplementary Figure 2. Maternal BDE-47 exposure and serum leptin levels in male offspring.
Levels of leptin in serum of male offspring. Data are expressed as mean ± SEM, n ≥ 8.

## Slide 3
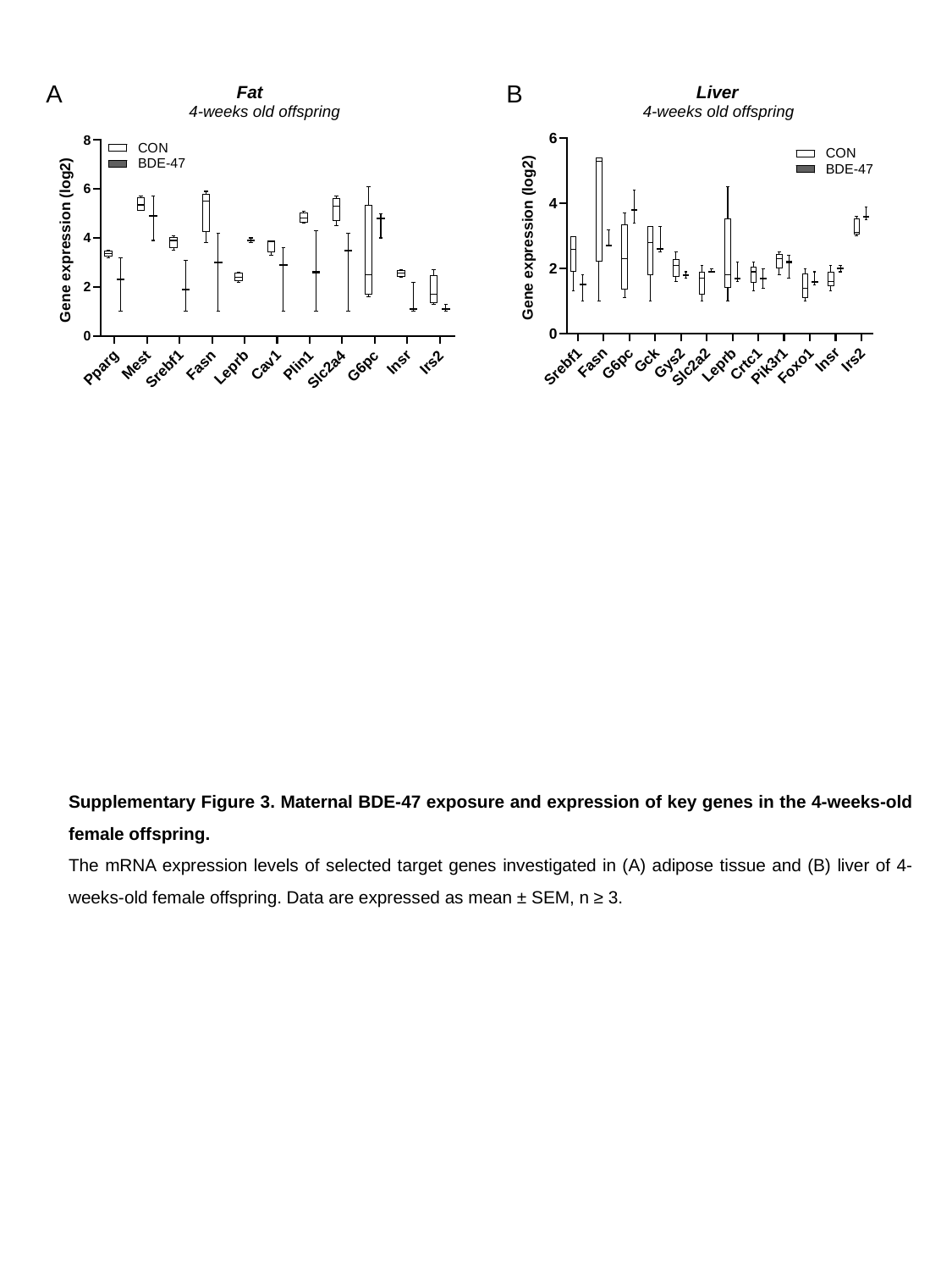

A
B
Fat
Liver
4-weeks old offspring
4-weeks old offspring
Supplementary Figure 3. Maternal BDE-47 exposure and expression of key genes in the 4-weeks-old female offspring.
The mRNA expression levels of selected target genes investigated in (A) adipose tissue and (B) liver of 4-weeks-old female offspring. Data are expressed as mean ± SEM, n ≥ 3.
